# Demographic and genetic factors in the recovery or demise of *ex situ* populations following a severe bottleneck in fifteen species of Hawaiian tree snails

**DOI:** 10.7717/peerj.1406

**Published:** 2015-11-12

**Authors:** Melissa R. Price, David Sischo, Mark-Anthony Pascua, Michael G. Hadfield

**Affiliations:** 1 Kewalo Marine Laboratory, Pacific Biosciences Research Center, University of Hawaiʻi at Mānoa, Honolulu, HI, USA; 2 Department of Natural Resources and Environmental Management, University of Hawaiʻi at Mānoa, Honolulu, HI, USA; 3 Division of Forestry and Wildlife, Department of Land and Natural Resources, Honolulu, HI, USA; 4 Biology Department, University of Hawaiʻi at Mānoa, Honolulu, HI, USA

**Keywords:** Endangered species, Captive-rearing, Population genetics, Extinction, Achatinellinae, Self-fertilization

## Abstract

Wild populations of endangered Hawaiian tree snails have declined precipitously over the last century due to introduced predators and other human impacts. Life history traits, such as very low fecundity (<5 offspring per year across taxa) and maturity at approximately four years of age have made recovery difficult. Conservation efforts such as *in situ* predator-free enclosures may increase survival to maturity by protecting offspring from predation, but no long-term data existed prior to this study demonstrating the demographic and genetic parameters necessary to maintain populations within those enclosures. We evaluated over 20 years of evidence for the dynamics of survival and extinction in captive *ex situ* populations of Hawaiian tree snails established from wild-collected individuals. From 1991 to 2006, small numbers of snails (<15) from fifteen species were collected from the wild to initiate captive-reared populations as a hedge against extinction. This small number of founders resulted in a severe bottleneck in each of the captive-reared populations. We identified key demographic parameters that predicted population recovery from this bottleneck. Species with captive populations that produced between two and four offspring per adult per year and had 20–50% of those offspring survive to maturity recovered to numbers above 100 individuals, and maintained viable populations following a decline that occurred between 2009 and 2014. Those populations that had less than two offspring per adult per year and less than 20% survival to maturity did not reach 100 individuals in captivity, and many of these populations died out during the recent decline. We suggest that small reductions in fitness may contribute to extirpation in taxa with inherently low fecundity, by keeping populations below a threshold number essential to long-term recovery. Future *ex situ* populations should be founded with no less than 15 adults, and maintained in conditions closely approximating the temperature and humidity of source locations to optimize fitness. Permanent translocations of wild populations for conservation purposes will be more likely to succeed with greater than 100 adults, and should be limited to locations with a similar climate to source locations.

## Introduction

Hawaiian tree snails display multiple life history traits such as very low fecundity (<5 offspring per year per adult, on average, across taxa) and maturity at approximately four years of age (Hadfield, Holland & Olival, 2004), that leave them vulnerable to human-influenced impacts. A high rate of extinction has followed centuries of habitat destruction, shell collecting and the introduction of predators such as rats, a predatory snail, and Jackson’s chameleons (Hadfield & Mountain, 1980; Hadfield, 1986; Hadfield, Miller & Carwile, 1993; Holland, Montgomery & Costello, 2009). Historical records indicate tree snail populations were once dense and abundant, occurring from sea level to at least 1,500 m elevation, but today only fragmented, small populations remain in high-elevation native forest, with many species listed as Endangered (US Fish and Wildlife Service, 2013) (Table 1).

**Table 1:** Hawaiian tree snail captive and wild population status and minimum remaining numbers. Minimum wild *N* is based on the minimum number of snails observed during surveys conducted within the last ten years.

Genus	Species	2014 captive total (*N*)	2014 captive adults (*N*)	Wild population status	Minimum wild N^*^
Population exceeded 100 individuals in captivity					
*Achatinella*	*fuscobasis*	124	35	Critically endangered	5
*Achatinella*	*lila*	183	51	Critically endangered	105
*Achatinella*	*livida*	25	3	Critically endangered	76
*Partulina*	*variabilis*	51	5	Critically endangered	51
Population never exceeded 100 individuals in captivity					
*Achatinella*	*apexfulva*	1	1	Extinct	–
*Achatinella*	*bulimoides*	10	1	Critically endangered	28
*Achatinella*	*decipiens*	2	1	Endangered	913
*Achatinella*	*fulgens*	2	0	Critically endangered	11
*Partulina*	*semicaranata*	6	2	Critically endangered	15
Population extirpated from captivity					
*Achatinella*	*sowerbyana*	–	–	Endangered	533
*Newcombia*	*cumingi*	–	–	Critically endangered	20
*Partulina*	*mighelsiana*	–	–	Critically endangered	63
*Partulina*	*perdix*	–	–	Critically endangered	Unknown
*Partulina*	*physa*	–	–	Critically endangered	Unknown
*Partulina*	*proxima*	–	–	Critically endangered	16

**Notes.**

* Hawaiʻi Department of Land and Natural Resources, Snail Extinction Prevention Program annual report 2013.

Recovery efforts, such as predator control, *in situ* predator-free enclosures, and *ex situ* propagation, may salvage some Hawaiian tree-snail populations. However, we have a limited understanding of which factors are most important to the long-term persistence of hermaphroditic species that survive severe bottlenecks. Two factors often used to assess the likelihood of persistence are fecundity and survival. Low fecundity or high mortality can impede recovery in long-lived species, which depend on high adult survivorship over a long life of reproduction to make up for delayed maturity (Gibbon et al., 2000).

Demographic elements interact with genetic factors to influence the fate of populations. Threatened species often have lower heterozygosity than extant relatives, indicating decreased adaptive potential and increased risk for inbreeding depression and extinction (Spielman et al., 2004). These dynamics have been well-studied theoretically, but few studies have documented empirical measures of real populations as they decline to extinction (Fagan & Holmes, 2006). Even fewer empirical studies have compared species that survived a severe bottleneck with close relatives that died out after a similar event.

**Figure 1:**
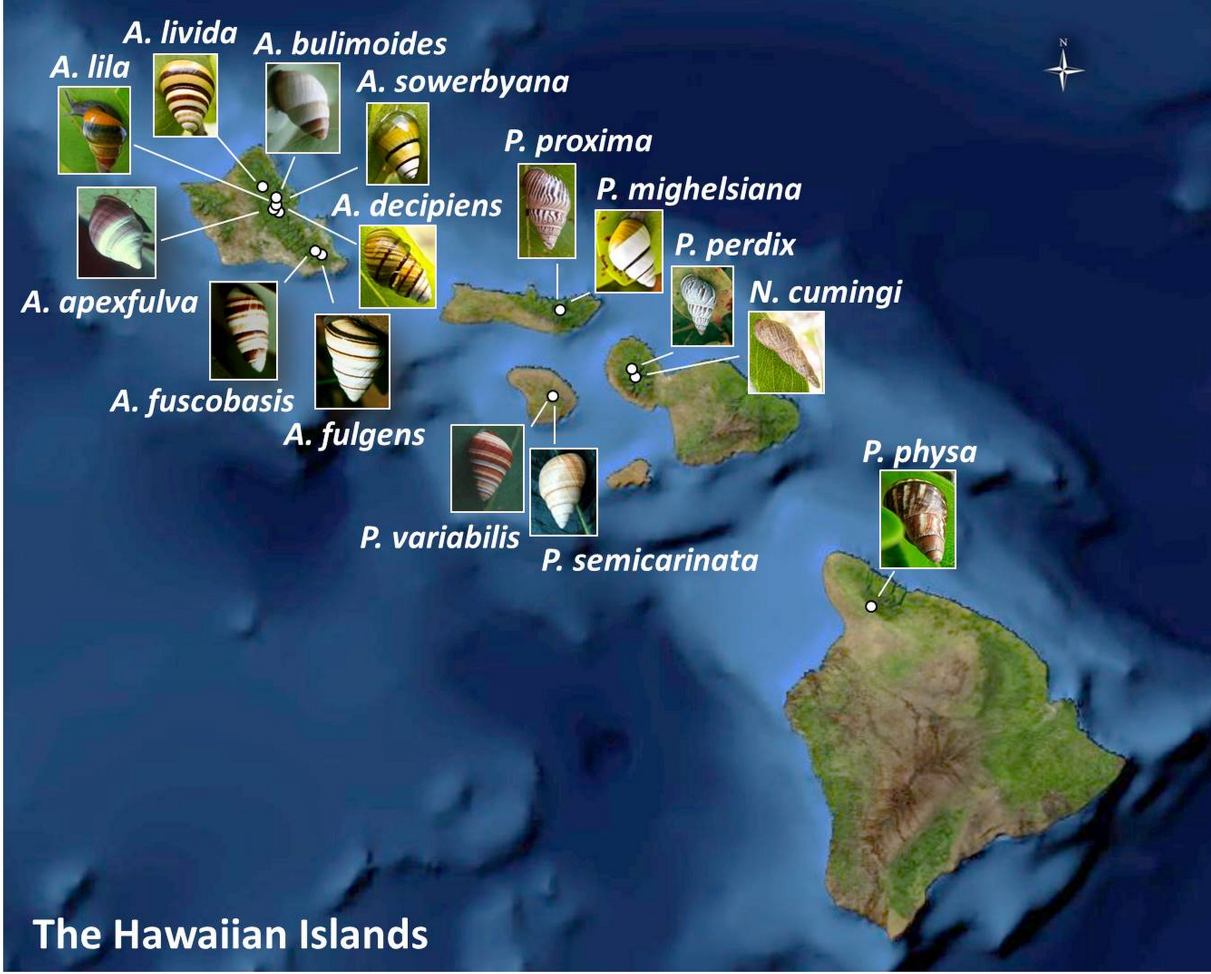
Collection sites for fifteen species of Hawaiian tree snails with populations in the captive-rearing facility at the University of Hawaiʻi at Mānoa. Species in the genus *Achatinella* are found only on Oʻahu. Species in the genus *Partulina* are found on Molokaʻi, Maui, Lanaʻi, and Hawaiʻi islands. Only one species in the genus *Newcombia*, from the island of Maui, was kept in the captive-rearing facility. Photo credits: *P. physa*: Melora Purell, other photos: authors’ own. Basemap sources: Esri, DigitalGlobe, GeoEye, i-cubed, USDA FSA, USGS, AEX, Getmapping, Aerogrid, IGN, IGP, swisstopo, and the GIS User Community.

Prompted by large declines observed around the year 2009 in captive populations of 15 species in the Hawaiian tree-snail subfamily Achatinellinae, including eight species in the genus *Achatinella*, one species in the genus *Newcombia*, and six species in the genus *Partulina*, we evaluated over 20 years of empirical evidence for the dynamics of survival and extinction. Captive populations were originally established for each of these species with less than 15 individuals collected from wild populations (Fig. 1), creating severe bottlenecks in the *ex situ* populations. We evaluated demographic measures over time within species to identify species-specific patterns across generations correlated with recovery. Next, fitness measures were compared among three groups (Table 1): (1) species that exceeded 100 individuals in captivity; (2) species that did not exceed 100 individuals in captivity; and (3) species that have since died out. Additionally, genetic influences on survival and population persistence were evaluated for four species, and compared with results from previous studies of two of the most successfully-reared tree snail species in the same lab (Price & Hadfield, 2014; Sischo et al., in press) to potentially identify trends across species.

## Materials and Methods

### Captive population husbandry

The captive-breeding facility for Hawaiian tree snails at the University of Hawaiʻi at Mānoa was initiated in 1991. Small numbers of snails (<15) were used to found captive populations between 1991 and 2006 (Table 2). Adults were preferentially collected when possible, but the ages of founders varied among species. Adults were identified by a “lipped” shell aperture, or a thickened edge of the shell opening, indicating sexual maturity (Kobayashi & Hadfield, 1996). The relatedness of the founding individuals of each species was unknown, but they were often collected over relatively small distances (less than 10 m). For two species, *A. fulgens* and *A. sowerbyana*, founders were collected from multiple locations, and the resulting populations were kept in separate cages until populations declined below a combined total of ten individuals, at which point a single combined population was established for each species.

**Table 2:** Demographic overview for achatinellid Hawaiian tree snails housed in the University of Hawaiʻi at Mānoa Endangered Tree Snail Captive Rearing Facility. With limited numbers remaining in the wild, captive populations were founded with a small number of snails (*N* = 2–13). Snails of two species, *Achatinella fulgens* and *A. sowerbyana*, came from different locations and were maintained as separate populations in captivity. Generation time (*T*) for each species of Hawaiian tree snail in captivity is according to the equation *M* − 1 + (1/(1 − *v*) (Nunney & Elam, 1994).

Genus	Species	Island of origin	Year population founded	Number of founders	*T*	Peak year adults	Peak adult *N*	Year *N* = 0
Exceeded 100 individuals in captivity								
*Achatinella*	*fuscobasis*	O‘ahu	1991	11	5.5	2004	149	
*Achatinella*	*lila*	Oʻahu	1997	7	5.6	2011	125	
*Achatinella*	*livida*	Oʻahu	1997	13	4.6	2011	34	
*Partulina*	*variabilis*	Lanaʻi	2000	9	6.1	2009	22	
Never exceeded 100 individuals in captivity								
*Achatinella*	*apexfulva*	Oʻahu	1997	10	8.7	2000	3	
*Achatinella*	*bulimoides*	Oʻahu	2006	7	4.4	2009	15	
*Achatinella*	*decipiens*	Oʻahu	1994	6	6.3	2006	12	
*Achatinella*	*fulgens*							
	Kului Gulch	Oʻahu	2006	4	5.9	2006	2	
	Kului holding	Oʻahu	2006	6	5.9	2007	4	
	Pia valley	Oʻahu	2006	8	5.9	2006	7	
	Pia east	Oʻahu	2006	2	5.9	2007	1	
*Partulina*	*semicaranata*	Lanaʻi	2000	8	5.7	2004	11	
Extirpated from captivity								
*Achatinella*	*sowerbyana*							
	Peahinaia	Oʻahu	1996	9	5.5	2002	6	2012
	Pulcherima	Oʻahu	1993	3	5.5	2005	7	2012
*Newcombia*	*cumingi*	Maui	1999	5	2.9	1999	5	2004
*Partulina*	*mighelsiana*	Molokaʻi	1994	7	3.7	2003	2	2007
*Partulina*	*perdix*	Maui	1999	3	4.9	2004	8	2012
*Partulina*	*physa*	Hawaiʻi	1995	3	5.3	2010	5	2010
*Partulina*	*proxima*	Molokaʻi	1994	4	5.6	2005	7	2010

Cage environments were controlled for temperature and humidity, regulated to mimic average field conditions at elevations of 600–650 m on the island of O’ahu (Hadfield, Holland & Olival, 2004). Hawaiian tree snails consume diverse microbial biofilms on leaf surfaces in the wild (Hadfield, Holland & Olival, 2004; O’Rorke et al., 2015), so leafy branches were collected from the mountains and placed in cages when they were cleaned biweekly, along with agar-cultured, calcium-supplemented mold (*Cladosporium* sp.) originally isolated from a native snail-host plant (Hadfield, Holland & Olival, 2004). This diet was previously shown to either increase growth rate compared with wild populations (Kobayashi & Hadfield, 1996), or at least produce an equivalent growth rate (Hadfield, Holland & Olival, 2004).

During bi-weekly cage cleanings, demographic information was collected including births, deaths, and total numbers of juveniles (<1 year-old), subadults (between 1 year and lipped), and adults (snails with a lipped shell). Individual snails were not labeled or tracked, but at death, each snail was individually preserved in 95% ethanol in a tube labeled with the species, source cage, date of preservation (within 2 weeks of death), and shell length and width.

Using biweekly reports we compiled a database of births, deaths, numbers of juveniles, subadults, and adults, and the total number of snails in each cage. We also noted any suspected cases of self-fertilization, in which an isolated adult snail produced offspring. Tree snails may store sperm, as in other hermaphroditic snails (Jordaens, Dillen & Backeljau, 2007), so in cases where a snail reached maturity when other mature snails were present in the cage, then later gave birth to offspring after some time of being isolated, we were not able to discern whether it was due to long-term sperm storage or self-fertilization. However, in some cases snails were isolated before maturity, and in these cases we could be sure any births were due to self-fertilization.

Using preserved snail samples we compiled a database of life-history information for individual snails including shell length and width at death, cage name, and date of preservation. Birth dates and an approximate date of sexual maturity were estimated for each snail based on shell growth rates obtained from lab records and field studies (Hadfield & Mountain, 1980; Severns, 1981; Hadfield & Miller, 1989; Kobayashi & Hadfield, 1996).

### Tissue sample collection, DNA extraction and amplification

Tissue samples from four species, *A. apexfulva*, *A. fulgens*, *A. sowerbyana*, and *P. variabilis*, were collected from the entire set of preserved and living captive snails using sterile techniques. For live captive snails larger than 12 mm in shell length, samples were collected using nonlethal methods, cutting a very thin slice of tissue from the posterior tip of the foot, following the method of Thacker & Hadfield (2000), and storing in 100% ethanol for subsequent DNA extraction. This method has been used with both captive and wild snails for several decades, without causing any mortalities, as determined by observing captive snails post-sampling. DNA was extracted from tissue samples using a DNeasy Blood and Tissue Kit (Qiagen, Hilden, Germany) according to the manufacturer’s protocol, and eluted using two 200 µl washes of elution buffer (10 mM Tris-Cl, 0.5 mM EDTA). Live captive snails smaller than 12 mm were sampled by swabbing mucus from the snail’s body using a sterile polyester-tipped swab, and storing the tip of the swab in a sterile, dry tube at −20 °C until extraction. DNA was extracted from mucus using a QIAamp DNA Micro Kit (Qiagen, Hilden, Germany) according to the manufacturer’s protocol. Carrier RNA was added to the cell lysis buffer according to the manufacturer’s protocol for very small amounts of DNA, and DNA was eluted in 50 µl of elution buffer.

Each sample was genotyped at eleven microsatellite loci (Erickson & Hadfield, 2008; Sischo et al., in press), using the recommended amplification protocols for each primer set (Table 3). Bovine serum albumin (4 µM) was added to amplification reactions for preserved specimens that failed to amplify, likely due to the presence of hemocyanin derivatives (Akane et al., 1994). Genotyping reactions were performed by the Center for Genomic, Proteomic, and Bioinformatic Research at the University of Hawaiʻi at Mānoa. Amplification, genotyping and scoring were performed at least twice for each individual at all loci. Peakscanner version 1.0 (Applied Biosystems 2006) was used to visualize and identify alleles. Results from individual samples that failed to amplify at more than half of the loci were discarded.

**Table 3:** Four species of captive-reared Hawaiian tree snails genotyped at 11 loci. Two loci did not amplify across several of the species. The annealing temperature (*T_a_*), number of alleles (*N_A_*), microsatellite size range, and allelic richness (*A_R_*) are presented for each species.

		*Achatinella apexfulva*			*Achatinella fulgens*			*Achatinella sowerbyana*			*Partulina variabilis*		
Loci names (Genbank accession no.)	*T_a_*	*N_A_*	Size range (bp)	*A_R_*	*N_A_*	Size range (bp)	*A_R_*	*N_A_*	Size range (bp)	*A_R_*	*N_A_*	Size range (bp)	*A_R_*
AS812 (EU119381)^a^	61	5	229–239	3.9	5	229–253	2.8	11	223–245	4.0	7	227–245	4.2
AS32 (EU119382)^b^	61	7	198–237	5.6	6	201–261	3.7	3	198–204	2.1	7	156–210	2.7
AS46 (EU119383)	60	6	216–294	4.6	7	213–246	4.8	11	210–252	5.0	8	207–240	4.1
AS53 (EU119384)^a^	63	10	176–248	7.8	14	212–308	5.8	16	196–320	4.1	21	176–288	8.2
AS61 (EU119385)	58	9	166–241	6.3	13	157–208	6.0	11	166–211	4.1	8	154–226	5.9
AS62 (EU119386)	60	7	212–240	6.9	11	216–268	4.8	12	208–236	4.7	6	202–236	4.1
AS82 (EU119387)^b^	63	8	150–180	5.7	7	123–168	3.0	10	153–210	4.3	18	66–313	3.7
AS110 (EU119388)^c^	65	*	*	*	*	*	*	10	211–253	3.8	*	*	*
AS50 (KR872615)	54	7	363–393	6.7	6	339–411	4.8	10	300–396	4.2	7	342–389	3.3
AS96 (KR872616)	58	14	141–447	14.0	*	*	*	11	279–408	3.7	*	*	*
AS100 (KR872617)^a^	59	3	210–231	2.7	8	215–260	4.7	8	224–301	3.3	13	201–262	6.4

**Notes.**

For items with an asterisk (*), the locus did not amplify in that species (AS96), or was not attempted in that species due to a high percentage of null alleles in previous studies (AS110).

a Excluded from analyses for *A. apexfulva* due to a high frequency of null alleles.

b Excluded from analyses for *A. fulgens* due a high frequency of null alleles.

c Excluded from analyses for *A. sowerbyana* due a high frequency of null alleles.

### Environmental data

Shapefiles containing point data, including latitude, longitude, and elevation were obtained from recent surveys of Hawaiian tree snails (Hawaii State Department of Land and Natural Resources, pers. comm., 2013). Using the software program ArcGIS (ESRI 2013, version 10.2 for desktop), annual mean temperature (°C), mean monthly precipitation for all twelve months, and annual mean precipitation (mm) were obtained for each snail-occurrence point from publicly-available layers (http://rainfall.geography.hawaii.edu, downloaded August 15, 2013; Giambelluca et al., 2013). Several variables, including minimum and maximum mean temperature, monthly precipitation, and annual precipitation were then identified for each species across the known species’ range.

### Statistical analysis

#### Demographic trends

Fecundity, or the number of offspring per adult per year, was determined by dividing the total number of offspring born in a particular year by the number of adults living during that particular year. Survival to maturity was calculated as the proportion of offspring born in a given year that lived to maturity. Generation time (*T*) was calculated for each species (Table 2) according to the equation *M* − 1 + (1/(1 − *v*), where *M* is the mean maturation time, and *v* is the mean adult survival, or the number of adult snails surviving each year divided by the total number of adult snails alive during that year, averaged across years (Nunney & Elam, 1994).

All statistical analyses for demographic parameters were conducted with the statistical package JMP version 10.0 (2012). Trends across birth years were evaluated within species using linear regression. Fecundity among generations, within species, was evaluated using a Kruskal-Wallis rank sums analysis for species with three or more generations in captivity, or a Wilcoxon test, for species with only two generations in captivity. Next, demographic measures were compared among three groups: (1) species that exceeded 100 individuals in captivity; (2) species that did not exceed 100 individuals in captivity; and (3) species that have since died out. For these comparisons, analysis of variance (ANOVA) was employed for evaluating fecundity and survival to maturity, with a Tukey-Kramer HSD post-hoc test to identify which groups differed from each other.

Because the number of adults per cage may influence fecundity due to density and chemical cue concentration effects (Jordaens, Dillen & Backeljau, 2007), we used linear regression to evaluate whether there was a correlation between the number of adults within a cage and fecundity, both within species, for those that had multiple cages housing populations, and across all species.

#### Population genetics analysis

Genotypic data were collected from four achatinelline snail species (*Achatinella apexfulva, A. fulgens, A. sowerbyana, Partulina variabilis*) for comparison with two species assessed in two other studies (*A. lila*, Price & Hadfield, 2014; *A. fuscobasis*, Sischo et al., in press) to identify trends across species. Null-allele frequencies were assessed using the software Genepop (Rousset, 2008). Based on the results generated by Genepop, several loci were dropped from subsequent analyses due to a high probability of null alleles present, inconsistent amplification across samples, or very low levels of polymorphism (Table 3). The remaining loci amplified consistently across samples, were polymorphic, and had minimal null allele frequencies, and were therefore used for the remaining analyses.

The program Arlequin (Excoffier, Laval & Schneider, 2005) was used to calculate genetic measures, including the inbreeding coefficient (*F_is_*) and observed heterozygosity (*H_O_*). Rarified allelic richness was calculated using HP-RARE, to account for differences in sample size. Statistical tests were performed using the statistical package JMP version 10.0 (2012). Individual heterozygosity, calculated as the proportion of heterozygous loci, was arcsine transformed and compared among groups using a *t*-test or an ANOVA, as appropriate, depending on the number of groups compared. For the most recent generation in each species, whose members were not old enough to have reached sexual maturity at the time of sampling, genetic measures were compared between deceased and living snails, rather than between those that survived to maturity and those that did not.

#### Environmental data

Minimum and maximum mean temperature, monthly precipitation, and annual precipitation, were compared among three groups of species: (1) species that exceeded 100 individuals in captivity; (2) species that did not exceed 100 individuals in captivity; and (3) species that have since died out from captivity. Values were compared among groups using a Kruskal-Wallis (Rank Sums) non-parametric test, followed by a Tukey-Kramer HSD post-hoc test.

## Results

### Population trends

**Figure 2:**
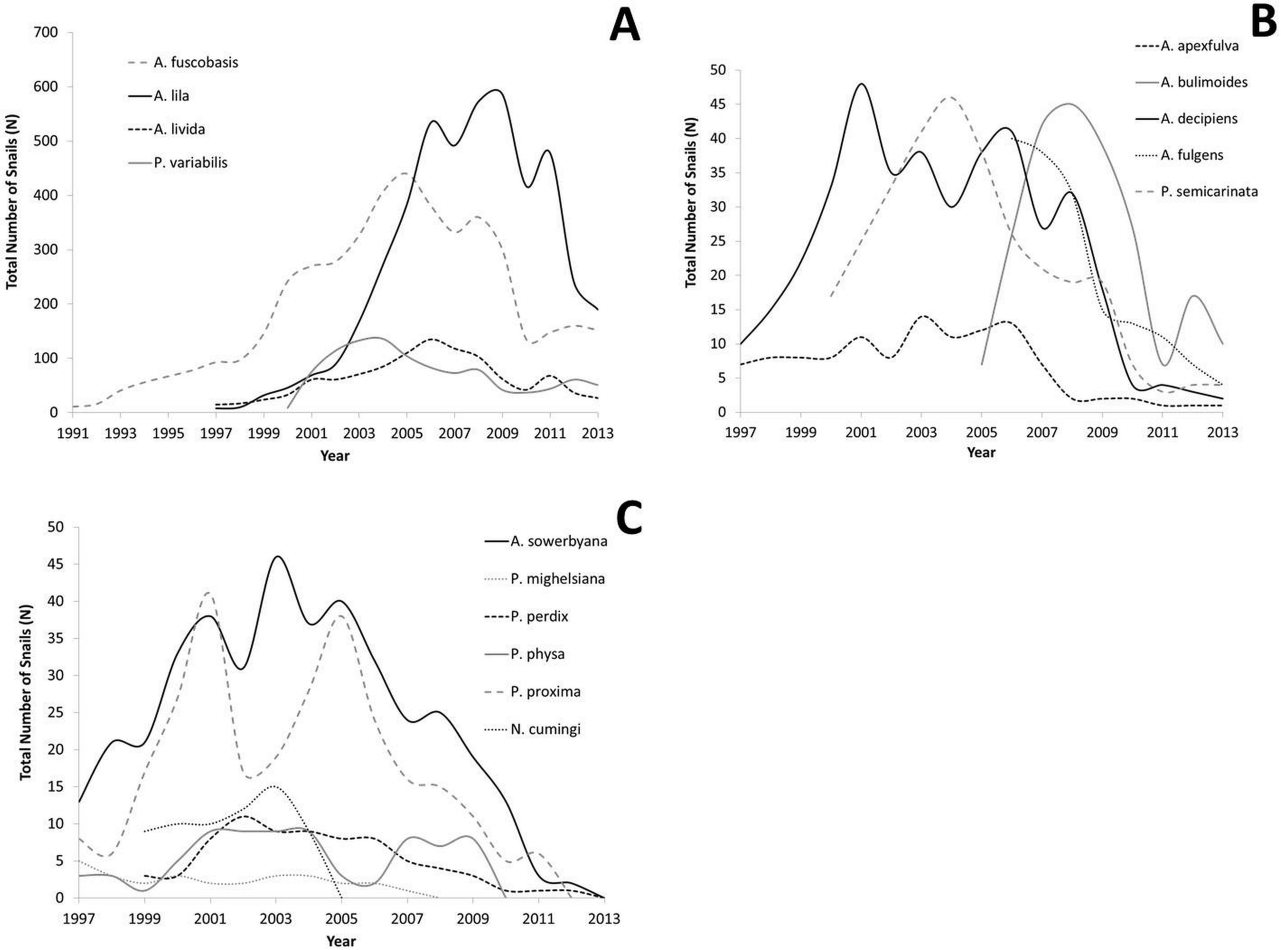
Population growth for each species over time. Captive populations were founded from 1991–2006 with a small number of individuals collected from the wild. (A) Species whose lab populations reached at least 100 individuals; (B) species whose lab populations did not exceed 100 individuals; (C) species whose lab populations have died out.

All captive tree snail populations in the captive-breeding laboratory at the University of Hawaiʻi have declined since reaching peak numbers, with the largest declines occurring since 2009 (Figs. 2A–2C). All lab populations that exceeded 100 snails have at least 25 snails remaining per species following the decline, and appear to have stabilized (Fig. 2A). All species whose peak population number was below 100 snails now have less than 10 snails (Fig. 2B), or have died out (Fig. 2C).

### Fecundity

**Figure 3:**
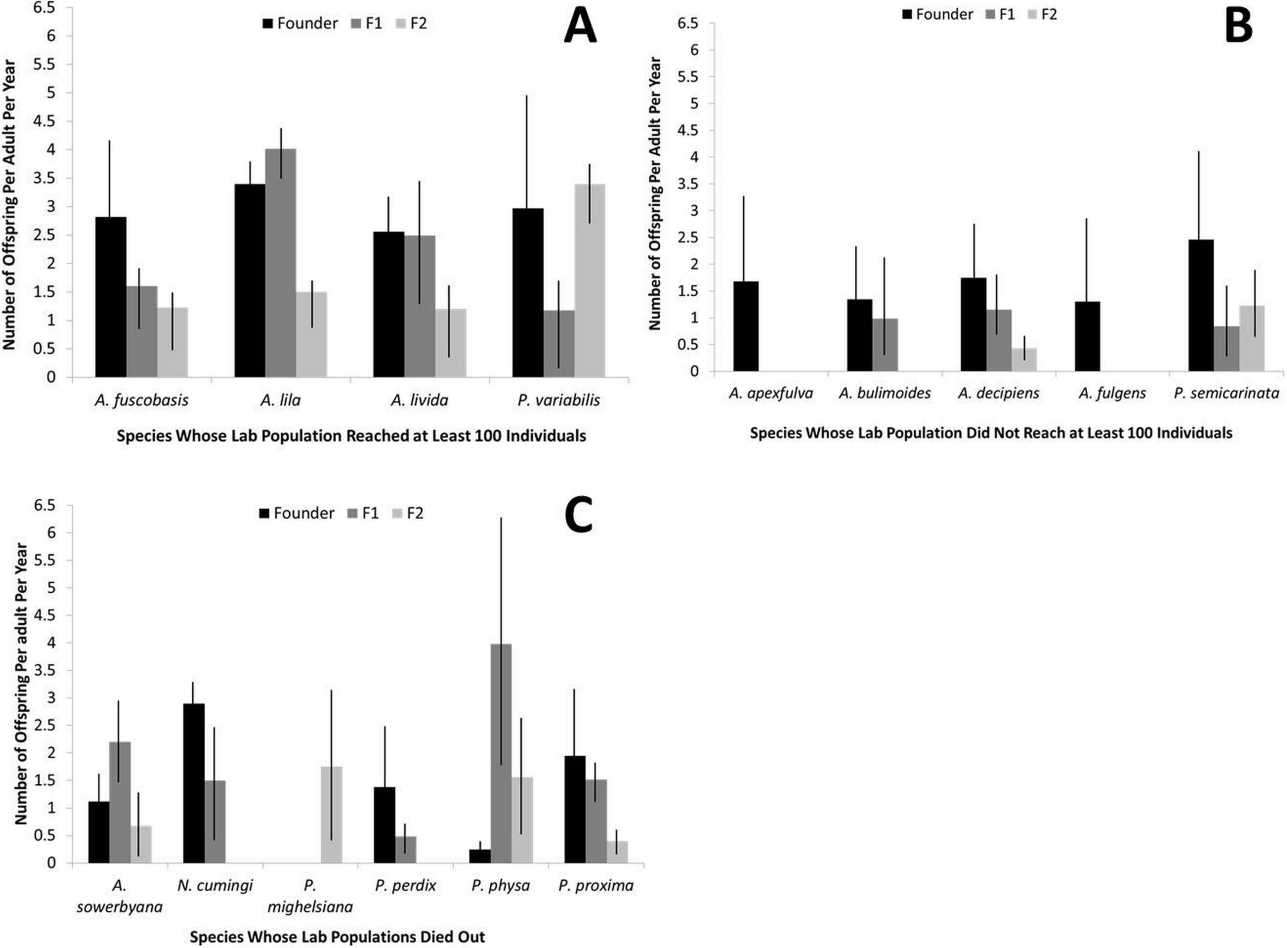
Fecundity by generation for each captive tree-snail population by species. Species that recovered to at least 100 individuals in the population (A) had higher fecundity, overall, than species that did not reach 100 individuals in the population (B) or died out (C).

Fecundity was significantly higher in the founding individuals of captive species that recovered to at least 100 individuals in captivity, compared with the two other groups (*r*^2^ = 0.16, *P* < 0.01; Fig. 3). When considered collectively, fecundity declined for all species over generations (*r*^2^ = 0.46, *P* < 0.01) and over birth years (*r*^2^ = 0.041, *P* < 0.001). The genera *Partulina* and *Achatinella* did not differ significantly in founder fecundity (*t* = 1.66, *P* = 0.10). Fecundity was not correlated with the number of adults in a cage (*r*^2^ = 0.0005, *P* = 0.66).

### Survival

**Figure 4:**
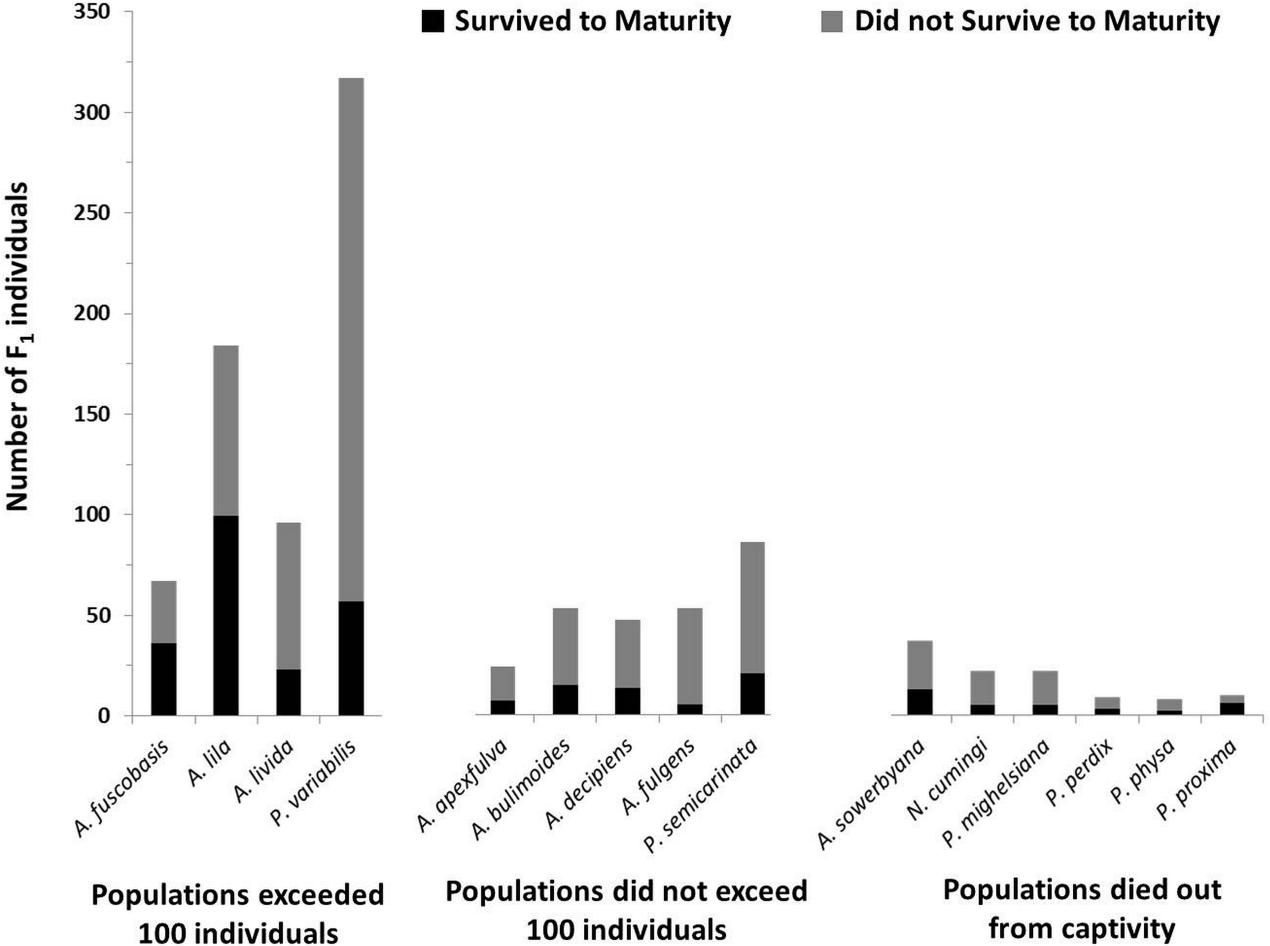
Number of offspring produced by the founding generation of each species. Black bars (bottom) represent the number of individuals that survived to maturity. Gray bars (top) represent the number of individuals born in the first generation that did not survive to maturity. Trends in fecundity and survival to maturity were significantly associated with species’ success or failure in the captive breeding program. The most successful species produced more than 2 offspring per adult per year, and had more than 20% of the offspring survive to maturity.

In addition to having more offspring per individual, offspring in the most successful species had significantly higher survival to maturity than species that did not exceed 100 individuals in captivity (*X*^2^ = 6.35, *P* = 0.012; Fig. 4), but did not significantly differ from species now extirpated from captivity (*X*^2^ = 0.047, *P* = 0.83).

### Genetic variation

**Figure 5:**
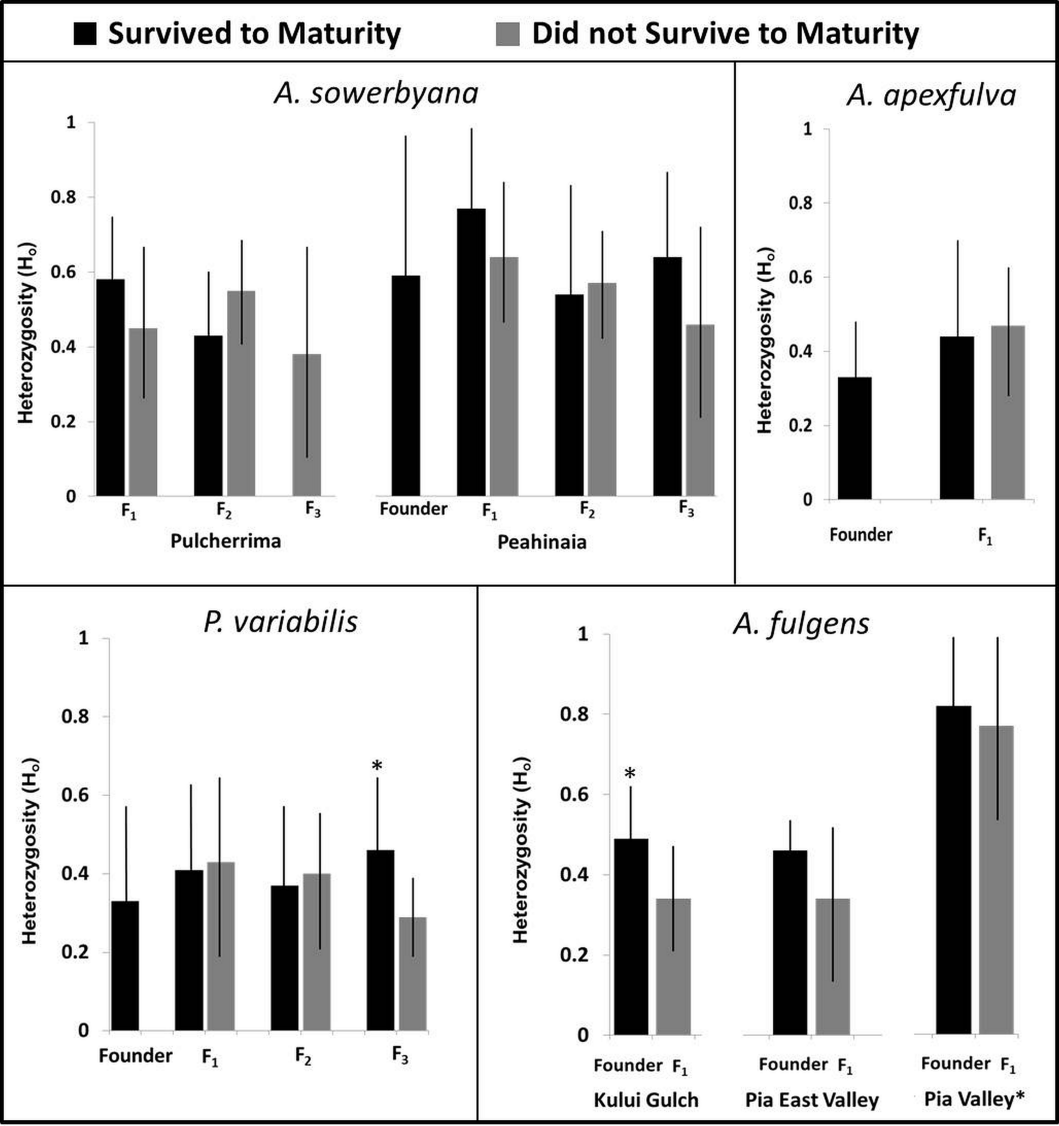
Heterozygosity compared with survival to maturity over generations in four species of captive-reared Hawaiian tree snails. Heterozygosity was significantly higher in individuals that survived to maturity in the third generation of *P. variabilis*, but not in other generations. The Pia Valley population of *A. fulgens* had significantly higher heterozygosity than the other populations of *A. fulgens*. All individuals in the *F*_1_ generation of *A. fulgens* died before maturity, except one individual in the Pia Valley population (not pictured). In the Kului Gulch population of *A. fulgens*, the *F*_1_ individuals had significantly lower heterozygosity than their parents. We were not able to obtain “founder” samples for the Pulcherrima population of *A. sowerbyana*.

Genetic samples were obtained from four species, *A. apexfulva, A. fulgens, A. sowerbyana*, and *P. variabilis* (Table 4), for comparison with published results from sister species *A. fuscobasis* (Sischo et al., in press) and *A. lila* (Price & Hadfield, 2014). Some correlations between heterozygosity and survival were observed within species (Fig. 5). Overall, heterozygosity was positively correlated with survival to maturity in captive-born individuals of *A. sowerbyana* (*r*^2^ = 0.019, *P* = 0.014), but there was no significant trend within generations. In the Kului Gulch population of *A. fulgens*, there was a significant decline in observed heterozygosity between the founders and the first generation of offspring (*t* = 2.71, *P* = 0.017), and none of the offspring survived to maturity. The other populations of *A. fulgens* also had declines in heterozygosity between the founder and *F*_1_ generations, but not significantly so, and only one individual in this species, from the Pia Valley population, survived to maturity. In the third generation of *P. variabilis*, surviving individuals had significantly higher heterozygosity than those that did not survive (*t* = 3.49, *P* < 0.001).

**Table 4:** Total number of captive snails (deceased and living), and genetic samples obtained for each species in the University of Hawaiʻi at Mānoa Endangered Tree Snail Captive-Rearing Facility. In this study, four species were genotyped at eleven loci, to compare with two species (*A. fuscobasis* and *A. lila*), which were examined in previous studies. The number of useable loci varied due to a high probability of null alleles for some loci in some species.

Genus	Species	Demographic (*N*)	Genetic samples (*N*)	No. Loci
*Achatinella*	*apexfulva*	35	28	6
*Achatinella*	*bulimoides*	72		
*Achatinella*	*decipiens*	143		
*Achatinella*	*fulgens*	56	49	7
*Achatinella*	*fuscobasis^a^*	1,536	342	7
*Achatinella*	*lila^b^*	2,018	533	6
*Achatinella*	*livida*	388		
*Achatinella*	*sowerbyana*	128	102	10
*Newcombia*	*cumingi*	36		
*Partulina*	*mighelsiana*	47		
*Partulina*	*perdix*	18		
*Partulina*	*physa*	49		
*Partulina*	*proxima*	109		
*Partulina*	*semicaranata*	124		
*Partulina*	*variabilis*	470	342	9

One species, *A. apexfulva*, did not show a similar correlation between heterozygosity and survival. Heterozygosity was slightly higher in the first generation of offspring than in the founders (Fig. 5), and individuals that did not survive to maturity had higher heterozygosity (0.48 ± 0.12), though not significantly so, than individuals that reached maturity (0.42 ± 0.20, *t* = 1.14, *P* = 0.26).

Inbreeding coefficients were significantly high in all of the most successful species in which we examined population genetics (Table 5). For one species that died out in the laboratory, *A. sowerbyana*, a decline in survival to maturity corresponded with an increase in the inbreeding coefficient over time. The first generation of adults from both populations of *A. sowerbyana* did not exhibit high inbreeding coefficients (*Fis*_Peahinaia(*F*1)_ = − 0.112, *P* = 0.87, *Fis*_Pulcherrima(*F*1)_ = 0.029, *P* = 0.44), but the second generation of adults did (*Fis*_Peahinaia (*F*2)_ = 0.197, *P* < 0.01, *Fis*_Pulcherrima(*F*1)_ = 0.238, *P* = 0.060).

**Table 5:** Inbreeding coefficients (*F*_is_) for captive-reared Hawaiian tree snails and individuals used to found these populations (Founders). All *F*_is_ values above 0.110 showed significant departure from Hardy-Weinberg expectations. Significant *F*_is_ values could be an indication of inbreeding or a high occurrence of self-fertilization in these simultaneous hermaphrodites.

Species	Founder *F*_is_	*n*	*F*_1_ *F*_is_	*n*	*F*_2_ *F*_is_	*n*	*F*_3_ *F*_is_	*n*
Exceeded 100 individuals in captivity								
*A. fuscobasis* ^a^	No data		0.419	25	0.348	171	0.409	109
*A. lila* ^b^	0.395	6	0.375	85	0.413	244	0.291	167
*P. variabilis*	0.425	10	0.147	213	0.113	81	0.135	44
Never exceeded 100 individuals in captivity								
*A. apexfulva*	0.400	3	0.328	25				
*A. fulgens*								
Kului Gulch	0.219	7	0.305	13				
Pia valley	0.032	6	0.099	17				
Extirpated from captivity								
*A. sowerbyana*								
Peahinaia	0.197	5	0.046	16	0.265	22	0.191	9
Pulcherrima	No data		0.132	15	0.102	25		

**Notes.**

a Sischo et al. (in press).

b Price & Hadfield (2014).

### Environment

**Figure 6:**
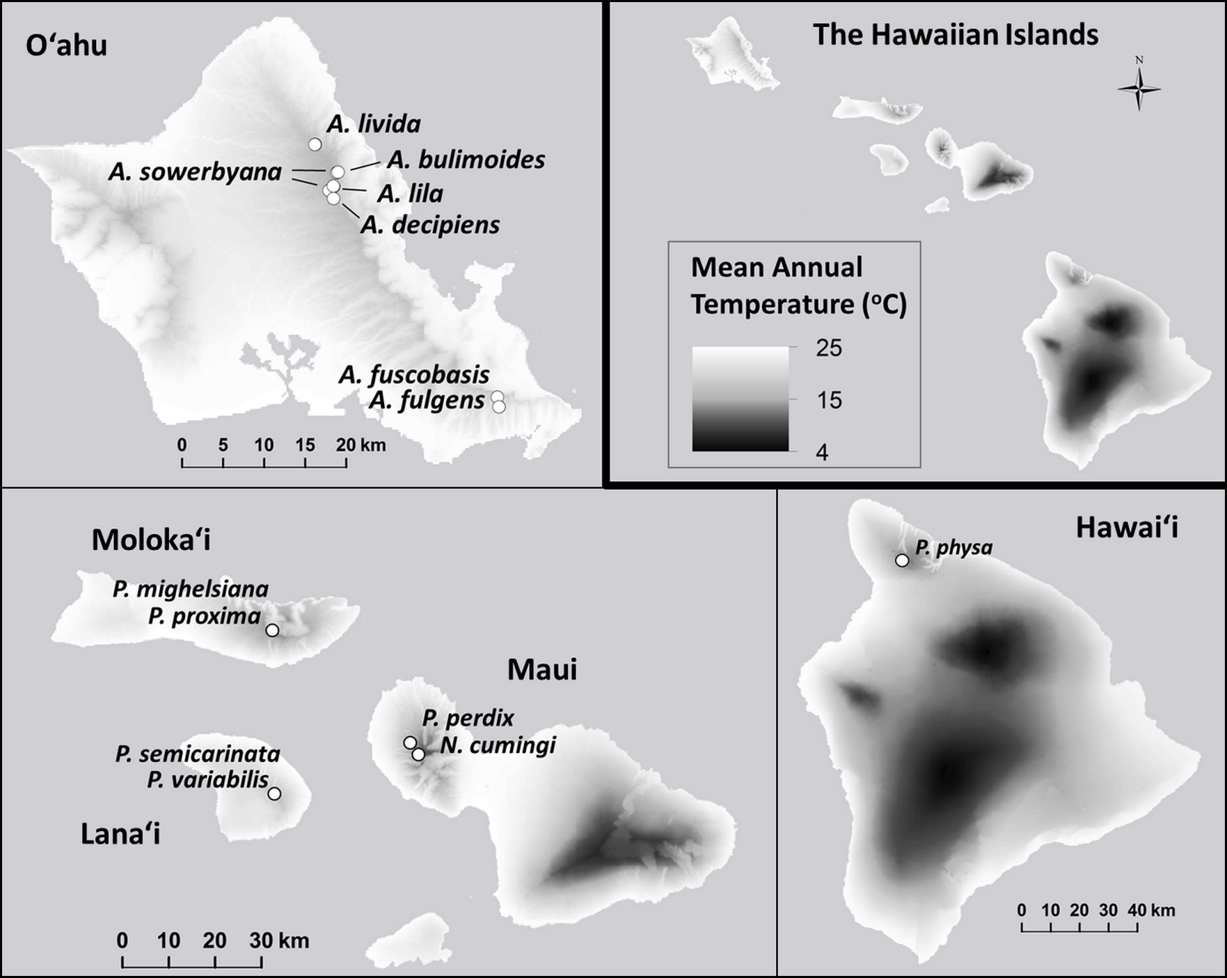
Mean annual temperature (°C) at the sites where wild snails were originally collected for the tree-snail captive-rearing facility at the University of Hawaiʻi at Mānoa. The mean annual temperature was significantly lower at the collection sites of snails with *ex situ* populations that did not successfully recover from the bottleneck that occurred at the founding of the captive populations. Data: Giambelluca et al. (2013).

**Figure 7:**
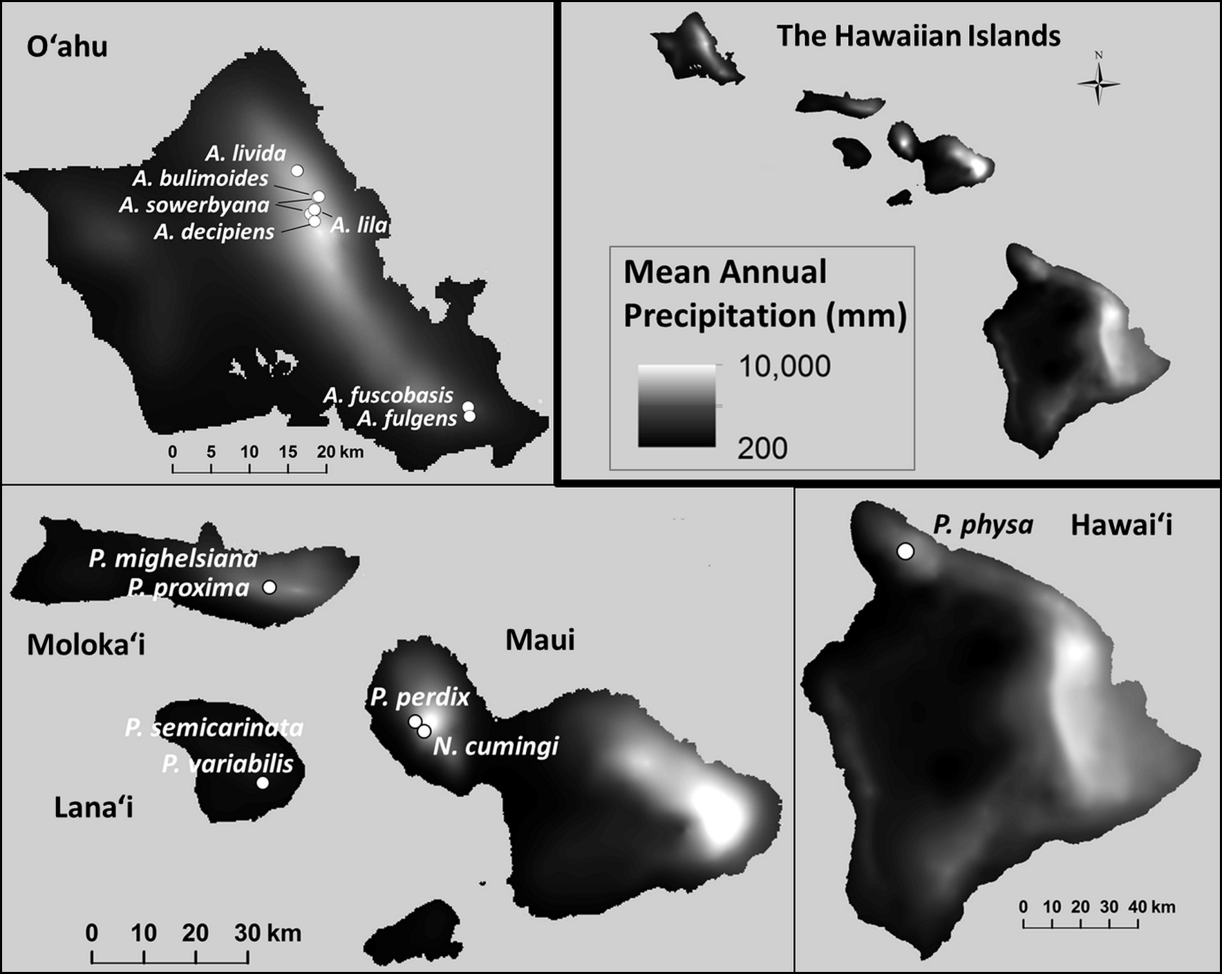
Mean annual precipitation (mm) at the sites where wild snails were originally collected for the tree-snail captive-rearing facility atthe University of Hawaiʻi at Mānoa. Precipitation was not correlated with population recovery. Data: Giambelluca et al. (2013).

Minimum and maximum mean annual temperatures (°C) within the species’ natural ranges were significantly lower in species that are now extirpated from captivity than in species that remain in captivity (${X}_{\mathrm{min}}^{2}=7.71$ *P*_min_ = 0.021; ${X}_{\mathrm{max}}^{2}=8.94$, *P*_max_ = 0.012; Fig. 6). Monthly (${X}_{\mathrm{monthly}\hspace{0.167em} \mathrm{min}}^{2}=0.075$, *P*_monthly min_ = 0.96; ${X}_{\mathrm{monthly}\hspace{0.167em} \mathrm{max}}^{2}=0.18$, *P*_monthly max_ = 0.91) and annual precipitation (${X}_{\mathrm{annual}\hspace{0.167em} \mathrm{min}}^{2}=0.067$, *P*_annual min_ = 0.96; ${X}_{\mathrm{annual}\hspace{0.167em} \mathrm{max}}^{2}=0.022$, *P*_annual max_ = 0.90; Fig. 7) varied substantially among and even within species, but was not predictive of survival in the captive breeding facility.

### Self-fertilization

Self-fertilization or long-term sperm storage was observed in multiple species from two achatinelline genera in captivity. Individuals of *A. fulgens, A. mustelina*, and *P. mighelsiana*, isolated for multiple years before maturity, gave birth to offspring. Individual *Achatinella decipiens, A. sowerbyana*, *Partulina physa*, and *P. semicarinata* gave birth after being isolated for 10 months or more.  In contrast, for several other species that died out in captivity, adults were isolated for years and never gave birth. The lone *A. apexfulva* that remains in the lab, the last known individual of its species, reached maturity in 2012, as indicated by forming a “lip” on the shell aperture, but has not produced any offspring.

## Discussion

Captive populations of fifteen species of Hawaiian tree snails experienced severe bottlenecks when small numbers of snails from each species were brought into a captive-rearing facility. Three factors were clearly predictive of recovery from this bottleneck: the number of founders, the fecundity of the founders, and survival to maturity in the first generation of offspring. Populations founded with less than 7 individuals never exceeded 100 individuals in captivity, and are now either in decline or have died out. Overall, populations of species that produced between two and four offspring per adult per year and had 20–50% survival to maturity were more likely to increase to a population size of at least 100 individuals. Those that had less than two offspring per year, of which less than 20% survived to maturity, were less likely to persist in captivity.

Some of the snail species maintained in the laboratory may be inherently more fecund due to evolutionary history. One of the taxonomic characters initially used to differentiate the genus *Partulina* from *Achatinella* was the presence of two offspring developing internally in *Partulina*, compared with one in *Achatinella* (Pilsbry & Cooke, 1912–1914). In our study, fecundity did not differ significantly between species in the genus *Achatinella* and those in *Partulina*, but it did vary within each genus. Even very closely related (Holland & Hadfield, 2004) and co-occurring species such as *P. variabilis* and *P. semicarinata* differed substantially in this measure. *Partulina variabilis* was much more fecund in captivity than *P. semicarinata*, and this correlates well with relative abundance observed during surveys of wild populations on Lanaʻi (USFWS report 2013, pers. obs., 2015). Fecundity data for wild snails are available for only two of the species in this study, limiting comparisons. In the wild, adult snails of *P. proxima* produced about 6.2 offspring per year (Hadfield & Miller, 1989), much higher than observed in the captive population, even in the founding snails (0–2 offspring per year). In contrast, a previous study found fecundity to be comparable between wild and *ex situ* populations of *P. redfieldii* (Hadfield, Holland & Olival, 2004).

In addition to discovering demographic indicators of population recovery from a severe bottleneck, we aimed to identify factors that led to the recovery of some species, or contributed to the decline of others. We suggest genetic diversity and environmental conditions likely played a role in fecundity and survival to maturity, and thus in determining whether or not a population recovered following the initial bottleneck. For four of six species examined in this and previous studies, heterozygosity correlated with demographic measures. Positive correlations between heterozygosity and survival to maturity were observed for *A. fulgens*, *A. sowerbyana, P. variabilis* and *A. lila* (this study; Price & Hadfield, 2014), highlighting the importance of maintaining genetic diversity and avoiding breeding among close relatives in endangered populations, particularly those raised *ex situ* (Theodorou & Couvet, 2015). Individuals of *A. lila* with higher heterozygosity were also more fecund (Price & Hadfield, 2014), suggesting fecundity might be improved by increasing genetic diversity within populations.

Self-fertilization has been observed in four species in the genera *Achatinella* and *Partulina*, and suspected in six additional species (this study; Kobayashi & Hadfield, 1996), suggesting there may be a widespread ability to self-fertilize in this subfamily. However, the ability to self-fertilize may be linked with high inbreeding coefficients and decreased heterozygosity, similar to other hermaphroditic species of snails (reviewed in Jordaens, Dillen & Backeljau, 2007). High inbreeding coefficients were observed in all generations of *A. fuscobasis*, *A. lila*, and *P. variabilis* (Sischo et al., in press; Price & Hadfield, 2014; this study), which were all very successful in captivity. One explanation for these high inbreeding coefficients may be that these species have a high occurrence of self-fertilization, even when mates for outcrossing are available. However, isolation experiments were not performed in these species to confirm the ability to self-fertilize. Two species in which self-fertilization was observed, *A. fulgens* and *A. sowerbyana*, both had increases in the inbreeding coefficient over generations (Table 5). In contrast, for several other species that died out in captivity, adults were isolated for years and never gave birth. The lone *A. apexfulva* that remains in the lab, the last known individual of its species, reached maturity in 2012, as indicated by forming a “lip” on the shell aperture, but has not produced any offspring.

Several environmental factors appear to be associated with population recovery. Species that did not survive in captivity had significantly lower annual mean temperatures in their native ranges than species that are persisting in captivity, suggesting the temperature in the laboratory environmental chambers, set to cycle year-round in 12-hour periods between 16 °C and 20 °C (Hadfield, Holland & Olival, 2004) may not have been optimal for these species. Three of the four most successful species in the *ex situ* facility, *A. fuscobasis, A. lila*, and *A. livida*, are found at or near 650 m elevation (Data S1), where data were collected when determining optimal chamber temperature cycles. Many other species are found at higher or lower elevations, and temperatures in the environmental chambers may have been too high or too low for optimal reproduction. An experiment in 2001 showed increased fecundity for *A. apexfulva* when gradually warmed to a 20 °C and 24 °C 12-hour alternating cycle (Hadfield, Holland & Olival, 2004). This species was historically found at lower elevations, but is now extirpated in the wild. Species’ natural ranges also vary substantially in precipitation, yet all captive populations were given the same levels of simulated precipitation. This may have significantly affected fitness in some species.

Despite substantial growth in the captive populations of some species during the first decade of captive-rearing Hadfield, Holland & Olival, 2004; Price & Hadfield, 2014; Sischo et al., in press, all populations have recently declined (Figs. 2A–2C), prompting several lines of investigation. Based on the observed demographic trends, we speculate that disease, and possibly changes in husbandry practices over time, may have contributed to the declines. Transmission electron micrographs of tissues from recently deceased snails were examined and determined to be without microsporidia (MGH, pers. obs., 2013), a parasite blamed for the extinction of another species of land snail kept by captive propagation at the London Zoo (Cunningham & Daszak, 1998). However, this does not exclude the possibility of infection with bacteria, viruses, or other disease-causing agents. In addition to causing direct mortality, disease or parasites may reduce fecundity in pulmonates (Cooper, Larson & Lewis, 1996).

High density in captive populations may lead to a decrease in fecundity if resources are limited (Ahmed et al., 1986; Richie, Hernandez & Rosa-Amador, 1966)), or an increase in fecundity due to a higher number of mating opportunities or cues (Rudolph & Bailey, 1985). We observed no correlation between fecundity and the number of adults per cage, suggesting that snail density was not responsible for the declines in fecundity over time.

### Implications for *in situ* and *ex situ* conservation efforts

Nonmarine mollusks have experienced very high extinction rates (Lydeard et al., 2004), yet little information is available to provide management targets for many of the species that remain. The present study provides several benchmarks for both *ex situ* and *in situ* populations of Hawaiian tree snails. Populations may be predicted to decline to extinction if numbers fall below two offspring per adult per year and 20% survival to maturity.

Our 20-year study provides empirical support for management-recovery targets of greater than 450 individuals for the remaining populations of rare Hawaiian tree snails in the wild, to buffer against stochastic threats such as disease or hurricanes. In this study, only those captive populations that recovered to more than 450 individuals prior to the recent declines were able to persist in numbers that suggest long-term stability. These recovery targets are necessary in wild populations to retain genetic diversity and maintain evolutionary potential as conditions change. The species with the most successful recovery in captivity, *Achatinella lila*, experienced a loss of allelic diversity over time (Price & Hadfield, 2014), perhaps due to widespread selective sweeps typical in captive populations as they adapt to conditions in captivity, or due to genetic drift, as is common in small populations (Frankham, 2012). For wild populations, increasing variability in temperature and precipitation may produce similar selective sweeps as they adapt to warmer, drier temperatures (Boutin & Lane, 2014; Charmantier & Gienapp, 2014; Crozier & Hutchings, 2014).

Invertebrates have historically received less funding than charismatic vertebrates, but are foundational to the survival of ecosystems and the maintenance of biodiversity. *Ex situ* programs for invertebrates must be maintained with the same rigor given to programs for charismatic species, with careful attention given to issues of disease, environmental conditions, and genetic stability. Prior to the recent declines, many species in the *ex situ* tree snail program at the University of Hawaiʻi were on a demographic trajectory toward recovery and success (Figs. 2A–2C). Today, more than twenty years after its initiation, only a few species are present in substantial numbers, and by many benchmarks, the *ex situ* effort for Hawaiian tree snails has not been successful. However, the source populations are now extinct for two species with sizeable *ex situ* populations remaining in captivity, *A. fuscobasis* and *A. lila* (Table 1). Furthermore, the *ex situ* effort has preserved the largest populations that exist today for these two species. A third species, *A. apexfulva*, no longer exists in the wild and has not been observed there for over 15 years. The one individual in the captive breeding facility represents the last of its species. In these three cases, snails exist today that would not have remained without the *ex situ* effort. Future efforts may be more successful if larger numbers of snails are brought to the captive-breeding facility to initiate captive populations, environmental-chamber settings closely mimic conditions in the wild for each individual species, fitness criteria are used to optimize husbandry, and genetic diversity is maintained.

## Supplemental Information

10.7717/peerj.1406/supp-1 Data S1 Environmental data for wild tree-snail populations Elevation, precipitation, and temperature data for wild snail populations. Precipitation and temperature data: Giambelluca et al. (2013).

10.7717/peerj.1406/supp-2 Table S1 Heterozygosity for four captive-reared populations of Hawaiian tree snails

10.7717/peerj.1406/supp-3 Table S2 Fecundity for fifteen species of captive-reared Hawaiian tree snails

10.7717/peerj.1406/supp-4 Table S3 Survival to maturity across generations for fifteen species of captive-reared Hawaiian tree snails

10.7717/peerj.1406/supp-5 Supplemental Information 1 Demographic data file for Achatinella apexfulva

10.7717/peerj.1406/supp-6 Supplemental Information 2 Demographic data file for Achatinella bulimoides

10.7717/peerj.1406/supp-7 Supplemental Information 3 Demographic data file for Achatinella decipiens

10.7717/peerj.1406/supp-8 Supplemental Information 4 Demographic data file for Achatinella fulgens

10.7717/peerj.1406/supp-9 Supplemental Information 5 Demographic data file for Achatinella fuscobasis

10.7717/peerj.1406/supp-10 Supplemental Information 6 Demographic data file for Achatinella lila

10.7717/peerj.1406/supp-11 Supplemental Information 7 Demographic data file for Achatinella livida

10.7717/peerj.1406/supp-12 Supplemental Information 8 Demographic data file for Achatinella sowerbyana

10.7717/peerj.1406/supp-13 Supplemental Information 9 Demographic data file for Newcombia cumingi

10.7717/peerj.1406/supp-14 Supplemental Information 10 Demographic data file for Partulina perdix

10.7717/peerj.1406/supp-15 Supplemental Information 11 Demographic data file for Partulina physa

10.7717/peerj.1406/supp-16 Supplemental Information 12 Demographic data file for Partulina proxima

10.7717/peerj.1406/supp-17 Supplemental Information 13 Demographic data file for Partulina variabilis

10.7717/peerj.1406/supp-18 Supplemental Information 14 Demographic data file for Partulina semicarinata

10.7717/peerj.1406/supp-19 Supplemental Information 15 Demographic data file for Partulina mighelsiana
