# Supplementary material for: Demographic and genetic factors in the recovery or demise of ex situ populations following a severe bottleneck in fifteen species of Hawaiian tree snails
Source: PeerJ. 2015 Nov 12;3:e1406. doi: 10.7717/peerj.1406 (PMC4647602; doi:10.7717/peerj.1406)
Supplement: Data S1 — Elevation, precipitation, and temperature data for wild snail populations. Precipitation and temperature data: Giambelluca et al. (2013). [file peerj-03-1406-s001.docx]

Elevation, precipitation, and temperature across the current extent of wild species ranges for species reared in the University of Hawai‘i at Mānoa Endangered Tree Snail Captive Rearing Facility. *Achatinella apexfulva* is not included in this table, as it is now extinct in the wild.

| Species | Min Elev (m) | Max Elev (m) | Min Month Prec (mm) | Max Month Prec (mm) | Min Annual Prec (mm) | Max Annual Prec (mm) | Min Annual Mean Temp (C) | Max Annual Mean Temp (C) |
| --- | --- | --- | --- | --- | --- | --- | --- | --- |
| Exceeded 100 individuals in captivity | | | | |  |  |  |  |
| *A. fuscobasis* | 650 | 650 | 90 | 254 | 1959 | 1959 | 20.4 | 20.4 |
| *A. lila* | 700 | 850 | 346 | 551 | 5101 | 5690 | 16.7 | 18.2 |
| *A. livida* | 700 | 800 | 299 | 514 | 4910 | 5227 | 17.4 | 18.1 |
| *P. variabilis* | 450 | 450 | 37 | 124 | 876 | 903 | 17.9 | 18.6 |
| Never exceeded 100 individuals in captivity | | | | |  |  |  |  |
| *A. bulimoides* | 575 | 775 | 339 | 545 | 5019 | 5690 | 17.3 | 18.6 |
| *A. decipiens* | 550 | 775 | 346 | 600 | 5101 | 6164 | 16.7 | 21.7 |
| *A. fulgens* | 375 | 400 | 81 | 239 | 1781 | 1845 | 21.0 | 21.4 |
| *P. semicaranata* | 450 | 450 | 37 | 124 | 876 | 903 | 17.9 | 18.6 |
| Extirpated from captivity | | | | | | | | |
| *A. sowerbyana* | 600 | 900 | 346 | 563 | 5100 | 5750 | 16.5 | 18.1 |
| *N. cumingi* | 750 | 925 | 233 | 408 | 3491 | 4352 | 16.7 | 17.7 |
| *P. mighelsiana* | 750 | 925 | 88 | 281 | 2232 | 2520 | 16.4 | 18.3 |
| *P. perdix* | 750 | 925 | 233 | 408 | 3491 | 4352 | 16.7 | 17.7 |
| *P. physa* | 900 | 1050 | 141 | 310 | 2943 | 3152 | 16.7 | 16.8 |
| *P. proxima* | 1050 | 1375 | 148 | 404 | 2760 | 3350 | 15.6 | 16.1 |
