# Supplementary material for: Demographic and genetic factors in the recovery or demise of ex situ populations following a severe bottleneck in fifteen species of Hawaiian tree snails
Source: PeerJ. 2015 Nov 12;3:e1406. doi: 10.7717/peerj.1406 (PMC4647602; doi:10.7717/peerj.1406)
Supplement: Table S1 [file peerj-03-1406-s002.docx]

| Species | Founder H_o_ | | *n* | F_1_ H_o_ | *n* | F_2_ H_o_ | *n* | F_3_ H_o_ | *n* | *X^2^/F* | *P* |
| --- | --- | --- | --- | --- | --- | --- | --- | --- | --- | --- | --- |
| Population exceeded 100 individuals in captivity | | | | | |  |  |  |  |  |  |
| *A. fuscobasis^a^* | | No data |  | 0.53±0.22 | 25 | 0.52±0.23 | 180 | 0.51±0.21 | 113 | 0.066 | 0.94 |
| *A. lila^b^* | | 0.43±0.20 | 6 | 0.44±0.03 | 85 | 0.38±0.01 | 244 | 0.39±0.02 | 167 | 1.45 | 0.23 |
| *P. variabilis* | | 0.33±0.24 | 10 | 0.43±0.20 | 213 | 0.40±0.18 | 81 | 0.41±0.18 | 44 | 1.12 | 0.34 |
| Population never exceeded 100 individuals in captivity | | | | | | |  |  |  |  |  |
| *A. apexfulva* | 0.33±0.21 | | 3 | 0.45±0.21 | 25 |  |  |  |  | 1.01 | 0.32 |
| *A. fulgens* |  | |  |  |  |  |  |  |  |  |  |
| Kului Gulch | 0.49±0.05 | | 7 | 0.34±0.05 | 13 |  |  |  |  | 2.71 | **0.017** |
| Pia Valley | 0.82±0.06 | | 6 | 0.77±0.04 | 17 |  |  |  |  | 0.60 | 0.56 |
| Pia East | 0.46±0.10 | | 2 | 0.34±0.09 | 4 |  |  |  |  | 1.11 | 0.34 |
| Population extirpated from captivity | | | | |  |  |  |  |  |  |  |
| *A. sowerbyana* |  | |  |  |  |  |  |  |  |  |  |
| Peahinaia | 0.70±0.012 | | 5 | 0.71±0.07 | 16 | 0.55±0.06 | 22 | 0.54±0.09 | 9 | 1.60 | 0.20 |
| Pulcherrima | No data | |  | 0.60±0.06 | 15 | 0.55±0.04 | 27 | 0.50±0.10 | 5 | 0.43 | 0.65 |

Heterozygosity by generation of species reared in the University of Hawai‘i at Mānoa Endangered Tree Snail Captive Rearing Facility. Heterozygosity significantly decreased over generations in one population of *A. fulgens*, but did not change across generations for the other populations.

^a^Sischo et al. 2015 (under review)

^b^Price and Hadfield (2014)
