# Supplementary material for: Demographic and genetic factors in the recovery or demise of ex situ populations following a severe bottleneck in fifteen species of Hawaiian tree snails
Source: PeerJ. 2015 Nov 12;3:e1406. doi: 10.7717/peerj.1406 (PMC4647602; doi:10.7717/peerj.1406)
Supplement: Table S2 [file peerj-03-1406-s003.docx]

Fecundity (number of offspring per adult per year) for each species of Hawaiian tree snail reared at the University of Hawai‘i at Mānoa Endangered Tree Snail Captive Rearing Facility. When considered collectively, fecundity declined for all species over generations (*r^2^* = 0.46, *P* < 0.01).

| Species | Founders  (mean±SD) | | F_1_  (mean±SD) | F_2_  (mean±SD) | F_3_  (mean±SD) | *r^2^ / X^2^* | *P* |
| --- | --- | --- | --- | --- | --- | --- | --- |
| All | 1.99±0.15 | | 1.80±0.11 | 1.40±0.11 | 1.05±0.24 | 0.046 | **<0.01** |
| Exceeded 100 individuals in captivity | | | | |  |  |  |
| *A. fuscobasis* | | No data | 1.60±0.57 | 1.23±0.74 | 0.98±0.73 | 5.92 | 0.052 |
| *A. lila* | | 3.40±0.65 | 4.02±0.59 | 1.50±0.65 |  | 4.34 | **0.036** |
| *A. livida* | | 2.56±0.90 | 2.49±1.26 | 1.20±1.15 |  | 4.47 | 0.11 |
| *P. variabilis* | | 2.97±2.17 | 1.18±1.07 | 3.40±0.43 |  | 7.33 | **0.026** |
| Never exceeded 100 individuals in captivity | | | | |  |  |  |
| *A. apexfulva* | | 1.68±1.51 |  |  |  |  |  |
| *A. bulmoides* | | 1.34±0.72 | 0.98±1.14 |  |  | 0.38 | 0.54 |
| *A. decipiens* | | 1.75±0.78 | 1.15±0.62 | 0.43±0.58 |  | 5.99 | 0.050 |
| *A. fulgens* | | 1.30±1.47 |  |  |  |  |  |
| *P. semicarinata* | | 2.46±1.35 | 0.84±0.71 | 1.23±0.72 |  | 10.84 | **<0.01** |
| Extirpated from captivity | | | |  |  |  |  |
| *A. sowerbyana* | | 1.12±0.48 | 2.20±0.86 | 0.68±0.78 | 0.67±1.15 | 12.61 | **<0.01** |
| *N. cumingi* | | 2.90±1.41 | 1.50±1.14 |  |  | 1.33 | 0.25 |
| *P. mighelsiana* | | No data | No data | 1.75±1.77 | 3.25±2.48 | 2.71 | 0.26 |
| *P. perdix* | | 1.38±1.57 | 0.48±0.44 |  |  | 2.71 | 0.26 |
| *P. physa* | | 0.25±0.35 | 3.98±2.36 | 1.56±1.30 |  | 7.01 | 0.072 |
| *P. proxima* | | 1.95±1.42 | 1.52±0.33 | 0.40±0.39 |  | 7.03 | 0.071 |
