# Supplementary material for: Demographic and genetic factors in the recovery or demise of ex situ populations following a severe bottleneck in fifteen species of Hawaiian tree snails
Source: PeerJ. 2015 Nov 12;3:e1406. doi: 10.7717/peerj.1406 (PMC4647602; doi:10.7717/peerj.1406)
Supplement: Table S3 [file peerj-03-1406-s004.docx]

Survival to maturity by generation for captive snails born in the University of Hawai‘i at Mānoa Endangered Tree Snail Captive-Rearing Facility.

| Species | F_1_ Survival to Maturity | F_1_ Total  (N) | F_2_ Survival to Maturity | F_2_ Total (N) | F_3_ Survival to Maturity | F_3_ Total (N) | *X^2^* | *P* |
| --- | --- | --- | --- | --- | --- | --- | --- | --- |
| Exceeded 100 individuals in captivity | | | | | |  |  |  |
| *A. fuscobasis* | 0.54 | 67 | 0.52 | 359 | 0.32 | 822 | 48.9 | **<0.0001** |
| *A. lila* | 0.54 | 184 | 0.50 | 932 | 0.13 | 354 | 153.8 | **<0.0001** |
| *A. livida* | 0.24 | 96 | 0.27 | 212 | 0.35 | 23 | 1.14 | 0.57 |
| *P. variabilis* | 0.18 | 317 | 0.19 | 88 |  |  | 0.13 | 0.72 |
| Never exceeded 100 individuals in captivity | | | | |  |  |  |  |
| *A. apexfulva* | 0.30 | 24 |  |  |  |  |  |  |
| *A. bulimoides* | 0.28 | 53 |  |  |  |  |  |  |
| *A. decipiens* | 0.28 | 47 | 0.10 | 63 | 0.04 | 27 | 10.3 | **<0.01** |
| *A. fulgens* | 0.10 | 53 |  |  |  |  |  |  |
| *P. semicarinata* | 0.24 | 86 | 0.36 | 22 |  |  | 1.27 | 0.26 |
| Extirpated from captivity | | |  |  |  |  |  |  |
| *A. sowerbyana* | 0.35 | 37 | 0.25 | 69 | 0.09 | 23 | 5.32 | 0.070 |
| *N. cumingi* | 0.23 | 22 | 0.21 | 14 | 0 | 1 | 0.29 | 0.86 |
| *P. mighelsiana* | 0.23 | 22 | 0.067 | 15 | 0.50 | 2 | 3.03 | 0.22 |
| *P. perdix* | 0.33 | 9 | 0.22 | 9 |  |  | 0.28 | 0.60 |
| *P. physa* | 0.25 | 8 | 0.26 | 19 | 0.46 | 11 | 1.38 | 0.50 |
| *P. proxima* | 0.60 | 10 | 0.27 | 26 | 0.18 | 62 | 8.43 | **0.015** |
